# Changes in technique throughout a 1500-m speed skating time-trial in junior elite athletes: Differences between sexes, performance levels and competitive seasons

**DOI:** 10.1371/journal.pone.0237331

**Published:** 2020-08-21

**Authors:** Inge K. Stoter, Florentina J. Hettinga, Egbert Otten, Chris Visscher, Marije T. Elferink-Gemser

**Affiliations:** 1 Center for Human Movement Sciences, University Medical Center Groningen, University of Groningen, Groningen, The Netherlands; 2 Centre of Sport and Exercise Science, School of Biological Sciences, University of Essex, Colchester, United Kingdom; 3 Department of Sport, Exercise and Rehabilitation, Faculty of Health and Life Sciences, Northumbria University, Newcastle upon Tyne, United Kingdom; Norwegian University of Science and Technology, NORWAY

## Abstract

Speed skating is a technical endurance sport. Still, little is known about technical changes in junior speed skaters. Therefore, changes in technique throughout a 1500-m time-trial of elite junior speed skaters is investigated to explore differences between sexes, performance levels and competitive seasons. At (inter)national 1500-m competitions, knee and push-off angles were obtained for 120 elite junior speed skaters (56 female, 64 male, age 17.6±1.1 years) per lap at 250m (lap 1), 650m (lap 2), 1050m (lap 3) and 1450m (lap 4). Additionally, 1500m end-times and lap-times were obtained to divide skaters in faster and slower performance groups and to analyze pacing behavior. Fifteen skaters (8 female, 7 male, age 17.3±1.5 years) were measured again after 1.6±0.6 years. (Repeated measures) ANOVAs were used for statistical analyses (p<0.05). ICC, determined in a pilot study, was 0.55 for knee and 0.76 for push-off angles. Elite junior speed skaters increased their knee angles throughout the race (p<0.005), regardless of sex (p = 0.110) or performance level (p = 0.714). Push-off angles increased from lap 1–3 (p<0.001), in which men showed a larger decay than female skaters (p<0.05), this holds for both performance groups (p = 0.103). Faster skaters had smaller knee and push-off angles than slower skaters (p<0.05). Males showed smaller body angles than females (p<0.001). Faster male and female skaters showed a relative slower start and faster lap 3 compared to slower skaters (p<0.05). Development over competitive seasons showed a shift towards smaller push-off angles (p = 0.038) and less decay in knee angles from lap 2–3 (p = 0.026). The present study shows that technique throughout the 1500m deteriorates. Deterioration in technique is regardless of performance level, even with different pacing behaviors. Differences between sexes were found for push-off angles. The longitudinal development suggests changes in technique towards senior level and highlights the importance of studying juniors separate from seniors.

## Introduction

**Fig 1:**
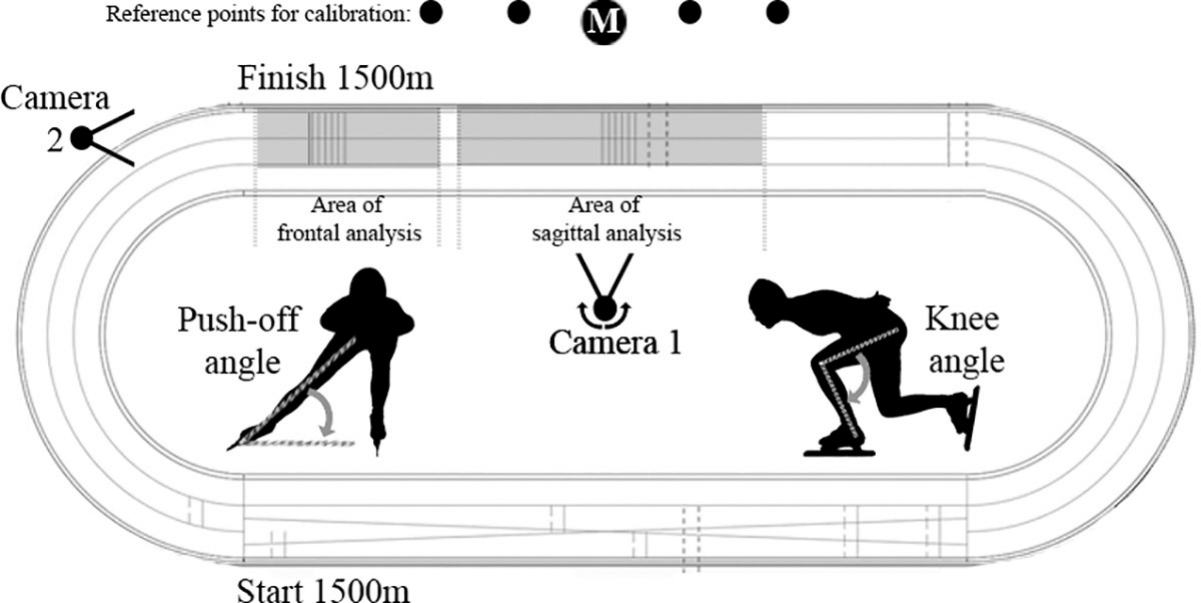
Measurement set-up to obtain knee angles (sagittal plane) and push-off angles (frontal plane) of the speed skaters. M represents the main reference point for the rotatable camera 1.

Speed skating is a peculiar sport in which the technique is characterized by a crouched position and a sideward push-off needed to move forward (Fig 1). How far elite junior speed skaters already resemble Olympic skaters in this respect is hardly known and topic of the present study. The crouched position with small knee angles is necessary to reduce aerodynamic resistance and extend push-off length [1–4]. However, when speed skaters fatigue, they typically show an increase in knee angles and push-off angles throughout the race, which is disadvantageous for the aerodynamics of the skaters and the efficiency of speed skating [5–8]. On the other hand, increasing knee angles might be beneficial for the oxygen delivery to the working leg muscles, as low knee angles in combination with high quasi-isometric muscular forces appear to cause a restriction of blood flow [9, 10]. Previous literature calculated that when higher knee angles cause 1 sec/lap loss of velocity due to higher aerodynamic resistance at a velocity of 35 sec/lap, they win 1 sec/lap by an increase of blood flow to the working muscle [9]. Changes in knee and push-off angles are likely coupled as higher knee angles will make it harder to have small push-off angles, since the leg is already closer to extension in the gliding phase. This might result in an additional negative effect of the increasing knee angles on the speed skating performance.

Dealing with the biomechanical benefits and the physiological disadvantages of a crouched position plays an important role in speed skating [2]. The present study focuses on the 1500-m time-trial, which takes around two minutes. Velocities up to 60 km/h can be reached and 50% of the metabolic energy is contributed by the aerobic system for which oxygen delivery is essential [11]. The distribution of energy over the race, so called pacing, is important for 1500-m performance in junior and senior speed skating [12, 13]. The commonly shown pacing behavior in 1500-m speed skating is a fast start, followed by a decrease in velocity towards the end of the race [8, 12, 13]. It is shown that pacing behavior, and there with the distribution of energy over the race, develops during adolescence and that junior and senior pacing profiles are not similar [13]. Part of the energy distributed over a race goes to the maintenance of technique. With changes of pacing behavior from junior to senior level, it might be that the profile of changes in technique over a race also change during adolescence. This is to our knowledge not yet investigated.

Growth and maturation are of influence on body posture and performance development for junior speed skaters (age < 19 years) and therewith possibly also on technique and maintenance of technique [14, 15]. However, studies on changes in technique in speed skating were mainly done on senior speed skaters [7, 8]. Only one article was found on junior speed skaters, but this study was limited to 8 athletes, both male and female, at their end of adolescence, who performed at the highest international level [5]. The study did not include push-off angles, but showed a general increase of knee angles from 98 to 110 degrees with a standard deviation of around 10 degrees [5].

In the study on the technique of junior speed skaters no distinctions were made between sexes and performance level [5]. Research on senior speed skaters did show differences between sexes and performance level [4, 7, 16]. In the most recent study on senior speed skaters, males showed smaller push-off angles, than female speed skaters, whereas knee angles were similar between sexes [7]. Earlier studies however, did show higher knee angles for female senior speed skaters than for male senior speed skaters [4, 16]. Differences in technique between performance levels was only shown for push-off angles in senior speed skaters [7]. No solid conclusions on the changes in technique during speed skating in relation to sexes and performance level for especially junior speed skaters, can be made based on previous literature.

Therefore, the present study investigates the profile of changes in knee and push-off angles throughout a 1500-m time-trial of elite junior speed skaters and investigates differences between sexes and performance levels. Additionally, longitudinal development of maintenance of technique will be explored over competitive seasons to provide perspectives on the development of elite junior speed skaters towards senior level. Similar to senior skaters [7], it is hypothesized that female junior speed skaters will have higher push-off angles than male junior skaters and that better performing junior speed skaters have smaller push-off angles than their less performing peers. Knee angles are hypothesized to be similar across sexes, but might be better maintained by the older and better performing junior speed skaters.

## Materials and methods

### Participants

In total, 123 Dutch junior speed skaters (58 female, 65 male, age 17.6 ± 1.1 years), who skated at national and international level, participated in the study. Measurements were done during national and international public championships, hosted in the Netherlands. No informed consents were obtained as the skaters were filmed at a public event and performance data was publicly available online. This study and the waiver of the informed consent was approved by the local ethical committee (University Medical Center Groningen, NL) on number ECB/2012.01.31_2 and is in accordance with the Declaration of Helsinki.

### Experimental procedures

Measurements were performed at four national and international junior competitions from 2010–2014. All competitions were in The Netherlands on semi-indoor and indoor 400-m ice-tracks and held at the end of the competitive season (February/March). Skaters competed within the age-category under 17 (U17) when they were 15 or 16 years at the start (July 1^st^) of the speed skating season or in the age category under 19 (U19) when they were 17 or 18 years at the start of the season. Age at competition date was based on year and month of birth, which was obtained online [17]. The 1500-m season best time per skater was also extracted from online data [17]. Only the best Dutch junior speed skaters were selected to compete in the competitions. The 1500-m season best times of the junior speed skaters were on average 115.7 ±3.6% of the prevailing senior world record (WR), which was 111.79 seconds for female and 101.04 seconds for male skaters between 2010 and 2014 [18].

Split-times at 0–300 m, 300–700 m, 700–1100 m, 1100–1500 m, and 1500-m competition end-times were obtained from the organizing committee, who used electronic timing systems with an accuracy of one hundredth of a second. Performance was defined as the end-time presented as a percentage of the prevailing world record. Pacing behavior was defined as split-times converted to a percentage of the corresponding 1500-m competition end-time. This was done to compare skaters from different performance levels on their pacing behavior [13]. To exclude any irregular events, i.e. falling, speed skaters with an 1500-m competition end-time over 8% above their season best time, which was obtained online [17], were excluded from analyses (2 female and 1 male). The 8% was based on boxplot analyses of all competition end-times converted to a percentage of season best time. In total, 120 Dutch junior speed skaters (56 female, 64 male) were included for further analyses.

The present study measured knee and push-off angles to quantify technique, which is a commonly used method in speed skating [1, 4, 6–8, 16, 19]. The measurement set-up is illustrated in Fig 1. During the 1500 m, elite junior speed skaters were recorded with two high resolution camera’s (1920x1080 pixels) at 25 frames per second at the straight at 250m (lap 1), 650m (lap 2), 1050m (laps 3) and 1450m (lap 4).

#### Knee angles

Camera 1 was positioned on the middle of the ice-track, 25m from the inner lane, at a height of 1.35m with a fixed zoom focusing on the area of analysis of the sagittal plane (Fig 1). Skaters were followed by the camera from the beginning of the straight to the end of the straight to get a good focus on the moving skater, keeping the skater in the middle of the video.

When the viewing angle of the rotatable camera to the skaters was not perpendicular to the straight, and therewith not perpendicular with the direction of the skaters, knee angles were corrected for the off-set of the viewing angle of the camera. To define the viewing angle of the camera a main reference point (M) was marked right in front of the camera perpendicular to the straight and in line with M additional reference points were marked and measured to calibrate the camera (Fig 1). The off-set of the viewing angle of the camera was defined by obtaining the perpendicular distance of the camera to M and the distance from M towards the calibration point in line with the viewing angle of the camera (Fig 1). With these two distances a triangle of the viewing angle of the camera with the perpendicular line from the camera to M and the line of calibration can be made to calculate the viewing angle of the camera.

Knee angles were obtained when the skater was closest to the camera, with one leg in gliding phase and the other leg in recovery phase, see Fig 1. Per measurement five frames were used, the frame in which the skater was closest to the position illustrated in Fig 1 and the 2 frames before and after this moment in which the skater was still in gliding phase were used for analysis. The knee angle was obtained using the location of the hip (trochanter major), the knee (epicondylus lateralis) and the ankle (malleolus lateralis) of the gliding leg. Further analyses were done with the average of the five frames per measurement.

#### Push-off angles

Camera 2 was placed outside the 400-m ice-track in the extension of the middle-line of the straight, which separates the inner from the outer competition lane. The camera was set at a height of 1.35m, with a fixed zoom focusing on the area of analysis of the frontal plane (Fig 1). Camera 2 filmed the frontal plane of the approaching skater to record the push-off angles. The push-off angle is the angle between the push-off leg with the horizontal line at the end of the push-off phase just before the first instance a part of the skate leaves the ice, see Fig 1. The position of the push-off leg was obtained using the location of the front-tip of the skate and the hip of the extending leg. Push-off angles were obtained from two following strokes, left and right, at the end of the straight. Per stroke two frames were used to analyze the angle between the push-off leg and the horizontal. Further analyses were done with the average of the four frames.

The analysis of body angles was done with one observer per measurement. We used data from multiple championships and a total of four observers were involved in the current study. In a pilot study, the inter-rater reliability was determined using the same protocol on previous observations. Two observers rating the same 56 races of 56 individuals, defined the intraclass correlation coefficient (single measures) for push-off angles (ICC = .76) and knee angles (ICC = .55). For push-off angles this indicates a good ICC, for knee angles a moderate ICC [20].

#### Cross-sectional data

Per age-category and sex, the 120 skaters were divided into faster and slower performance groups, based on their 1500-m end-time. Skaters with a 1500-m end-time below the average of their age-category and sex in the present study (female: 15–16 yrs = 121.1±4,1%WR and 17–18 yrs = 120.6±4.3%WR and male: 15–16 yrs = 120.3±5.5%WR and 17–18 yrs = 117.1±2.7%WR) were assigned to the faster performance group (26 female, 37 male). The others were assigned to the slower performance group (30 female, 27 male).

#### Longitudinal data

Fifteen (8 female, 7 male) of the 120 skaters were measured twice in the above mentioned elite junior 1500-m competitions, with at least one year in between measurements. The two measurements of the 15 skaters were used to explore longitudinal development of changes in knee and push-off angles throughout the 1500 m in speed skating.

### Statistics

All statistical analyses were performed using SPSS version 25, and significance was set at p < 0.05. All data are presented as mean ± standard deviation (mean±SD), if not other mentioned.

#### Cross-sectional data

Descriptive statistics were computed using independent t-tests to find differences in 1500-m season best time, age at competition, and the relative time spend on four race segments between performance categories for female and male skaters separately, with Cohen’s d (d) representing effect sizes. Thresholds for small, moderate, large, very large and extremely large effect sizes were set at 0.2, 0.6, 1.2, 2.0 and 4.0, respectively [21].

Repeated measures analysis of variance (ANOVA) explored changes in knee and push-off angles throughout the 1500-m time-trial for 120 skaters, with laps as within subject factor and sex and performance group as between subject factors. When sphericity was assumed, Greenhouse-Geisser correction was applied. Contrasts (repeated) were used when main or interaction effects with laps were found.

#### Longitudinal data

Descriptive statistics were computed using a paired t-test to find differences between the first and second measurement for 1500-m season best time, 1500-m competition end-time and the relative time spend on four race segments, with Cohen’s d (d) representing effect sizes.

Repeated measures ANOVA was used for finding differences in the development of the 15 junior speed skaters regarding changes in knee and push-off angles within the race, with measurement (first or second) and laps (1–4) as within subject variables and sex as between subject variable. When sphericity was assumed, Greenhouse-Geisser correction was applied. Contrasts (repeated) were used when main or interaction effects with laps were found.

## Results

### Cross-sectional data

Season best times were faster in the faster group for both female and male skaters (p<0.001), see Table 1. Age at competition was no different for the faster and slower groups in male (p = 0.638) and female (p = 0.947) skaters. Compared to the slower group, the faster group showed a higher relative time spend on the 0-300-m section in female (p = 0.007) and male (p<0.001) skaters (Table 1). Relative time spend on the 300-700-m section was similar for both performance groups in women (p = 0.697) and men (p = 0.717). Relative time spend on the 700–1100 m was lower for the faster group (i.e., they were faster) for women (p = 0.036) and men (p = 0.001) than the slower group. Final race segment did not show differences between performance groups for female skaters (p = .311), but for male skaters the faster performance group was relatively faster in the 1100-1500-m race segment than the slower group (p = 0.048).

**Table 1:** Descriptive statistics (mean±SD) of 120 elite junior speed skaters with 1500-m season best time and age, 1500-m end-time and relative time spend on the four race segments of the 1500-m competition, presented per performance group for female and male separately.

	Female			Male		
	Faster	Slower	d	Faster	Slower	d
	(n = 26)	(n = 30)		(n = 37)	(n = 27)	
**1500-m season best time**	126.63	131.18**	-1.52	115.18	119.82**	-1.44
**seconds**	±3.58	±2.28		±2.77	±3.62	
**(Inter)national junior1500-m competition:**						
**Age**	17.4	17.4	0.0	17.9	17.7	0.19
**years**	±1.0	±1.2		±0.93	±1.2	
**1500-m end-time**	128.92	135.49**	-2.44	116.28	122.18 **	-1.92
**seconds**	±2.74	±2.64		±2.13	±3.78	
**Relative time 0–300 m**	21.60	21.29*	0.73	22.04	21.60 **	1.14
**% of end-time**	±0.44	±0.41		±0.35	±0.42	
**Relative time 300–700 m**	24.78	24.81	-0.1	24.69	24.66	0.10
**% of end-time**	±0.29	±0.33		±0.26	±0.35	
**Relative time 700–1100 m**	26.18	26.34*	-0.58	25.96	26.17*	-0.89
**% of end-time**	±0.32	±0.22		±0.23	±0.24	
**Relative time 1100–1500 m**	27.43	27.56	-0.28	27.31	27.57*	-0.49
**% of end-time**	±0.47	±0.46		±0.40	±0.63	

Effect sizes are presented in column ‘d’.

Differences between performance categories are indicated by * (p<0.05) and ** (p<0.001).

#### Knee and push-off angles

**Fig 2:**
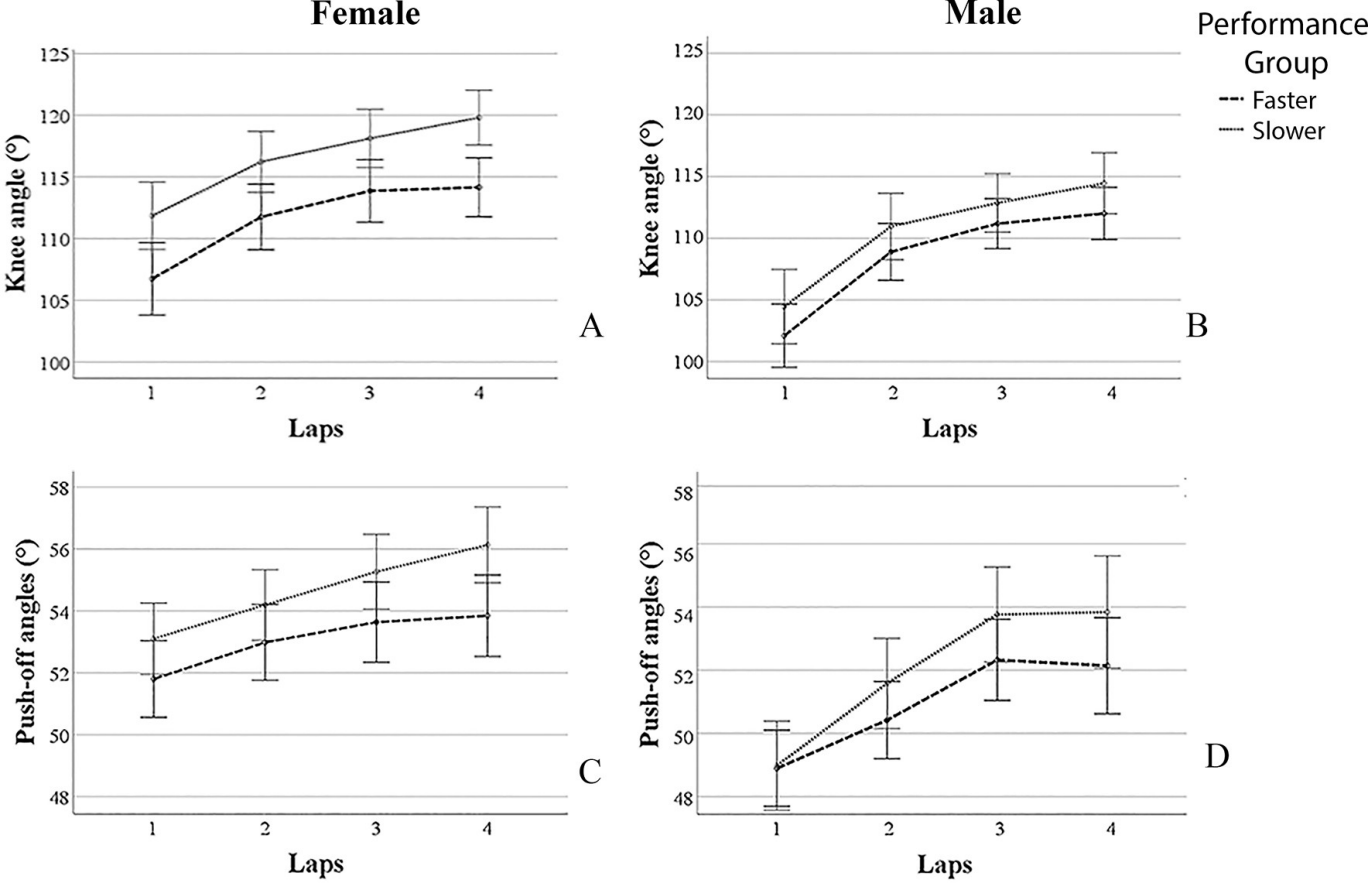
Changes in knee (A and B) and push-off (C and D) angles for the faster and slower performance groups, for female (A and C, n = 56) and male (B and D, n = 64) skaters separately. Error bars representing 95% confidence interval of the mean.

For knee angles, a main effect for laps (F(2.7,307.9) = 112.1, p<0.001) was found, with knee angles increasing from lap 1 to lap 2 (F(1,116) = 122.9, p<0.001), from lap 2 to lap 3 (F(1,116) = 22.7, p<0.001), and from lap 3 to lap 4 (F(1,116) = 4.9, p = 0.028). No interactions effects with laps were found, indicating similar decay in knee angles, regardless sex (p = 0.118) and performance level (p = 0.714). Between subject effects were found for sex (F(1,116) = 17.1, p<0.001) and performance level (F(1,116) = 10.6, p = 0.002). With 109.4± 5.7°, male speed skaters showed overall smaller knee angles than female skaters, who showed an average knee angle of 114.2± 6.5°. Also the faster group showed overall smaller knee angles than the slower group. No interaction effect of the between subject variables sex and performance level were found (p = 0.206). Changes in knee angles for female and male skaters can be seen in Fig 2A and 2B.

The analyses for push-off angles showed a main effect for laps (F(2.8,321.4) = 65.186, p<0.001), with push-off angles increasing from lap 1 to lap 2 (F(1,116) = 49.483, p<0.001) and from laps 2 to lap 3 (F(1,116) = 33.723, p<0.001), but not from lap 3 to lap 4 (p = 0.394). An interaction effect was found for laps*sex (F(2.8,321.4) = 5.681, p = 0.001), indicating that women had less decay in push-off angles than men from laps 1 to lap 2 (F(1,116) = 4.160, p = 0.044) and from lap 2 to lap 3 (F(1,116) = 5.548, p = 0.022). There was no interaction effect with performance level (p = 0.103). Between subject effects were found for sex (F(1,116) = 16.259, p<0.001) and performance level (F(1,116) = 5.241, p = 0.024). Male speed skaters showed overall smaller push-off angles (average = 51.4 ± 3.5°) than women (average = 53.9± 2.9°). Also the faster group showed overall smaller push-off angles than the slower group. No interaction effect of the between subject variables sex and performance level were found (p = 0.665). Changes in push-off angles for female and male skaters can be seen in Fig 2C and 2D.

### Longitudinal data

Descriptive statistics for the first and second measurement are provided in Table 2. Differences in the longitudinal data were found in corresponding 1500 m season best time (p = 0.003), with better season best times at an older age. No difference in 1500 m competition end-times (p = .120) or race segments, 0–300 m (p = .550), 300–700 m (p = .758), 700–1100 m (p = .511) and 1100–1500 m (p = .338), were found between the two measurements.

**Table 2:** Descriptive statistics (mean±SD) of first and second (1.6±0.6 years later) measurements of elite junior speed skaters (female, male and total), with 1500-m season best time and age, 1500-m end-time and relative time spend on the four race segments of the 1500-m competition.

	Female			Male			Total		
	(n = 8)			(n = 7)			(n = 15)		
Measurement	First	Second	d	First	Second	d	First	Second	d
**1500-m season best time**	128.30	125.32**	0.67	113.47	112.31	0.35	121.38	119.20 **	0.27
**seconds**	±5.02	±3.81		±3.12	±3.53		±8.68	±7.64	
**1500-m competition:**									
**Age**	17.0	18.6**	-1.14	17.6	19.1**	-1.15	17.3	18.9 **	-1.18
**years**	±1.5	±1.3		±1.4	±1.2		±1.5	±1.2	
**1500-m end-time**	131.50	129.50	0.49	114.66	114.08	0.22	123.64	122.33	0.15
**seconds**	±4.56	±3.47		±2.45	±2.8		±9.41	±8.55	
**Relative time 0–300 m**	21.66	21.60	0.12	22.10	22.03	0.40	21.87	21.80	0.16
**% of end-time**	±0.43	±0.56		±0.06	±0.24		±0.38	±0.48	
**Relative time 300–700 m**	24.72	24.77	-0.16	24.65	24.53	0.72	24.69	24,66	0.11
**% of end-time**	±0.38	±0.24		±0.11	±0.21		±0.28	±0.25	
**Relative time 700–1100 m**	26.23	26.16	0.26	25.88	25.85	0.15	26.07	26.02	0.18
**% of end-time**	±0.23	±0.31		±0.24	±0.14		±0.28	±0.29	
**Relative time 1100–1500 m**	27.39	27.47	-0.16	27.36	27.59	-0.80	27.38	27.52	-0.35
**% of end-time**	±0.54	±0.44		±0.25	±0.32		±0.42	±0.38	

Effect sizes are presented in column ‘d’.

Differences between first and second measurements are indicated by ** (p<0.001).

#### Knee and push-off angles

**Fig 3:**
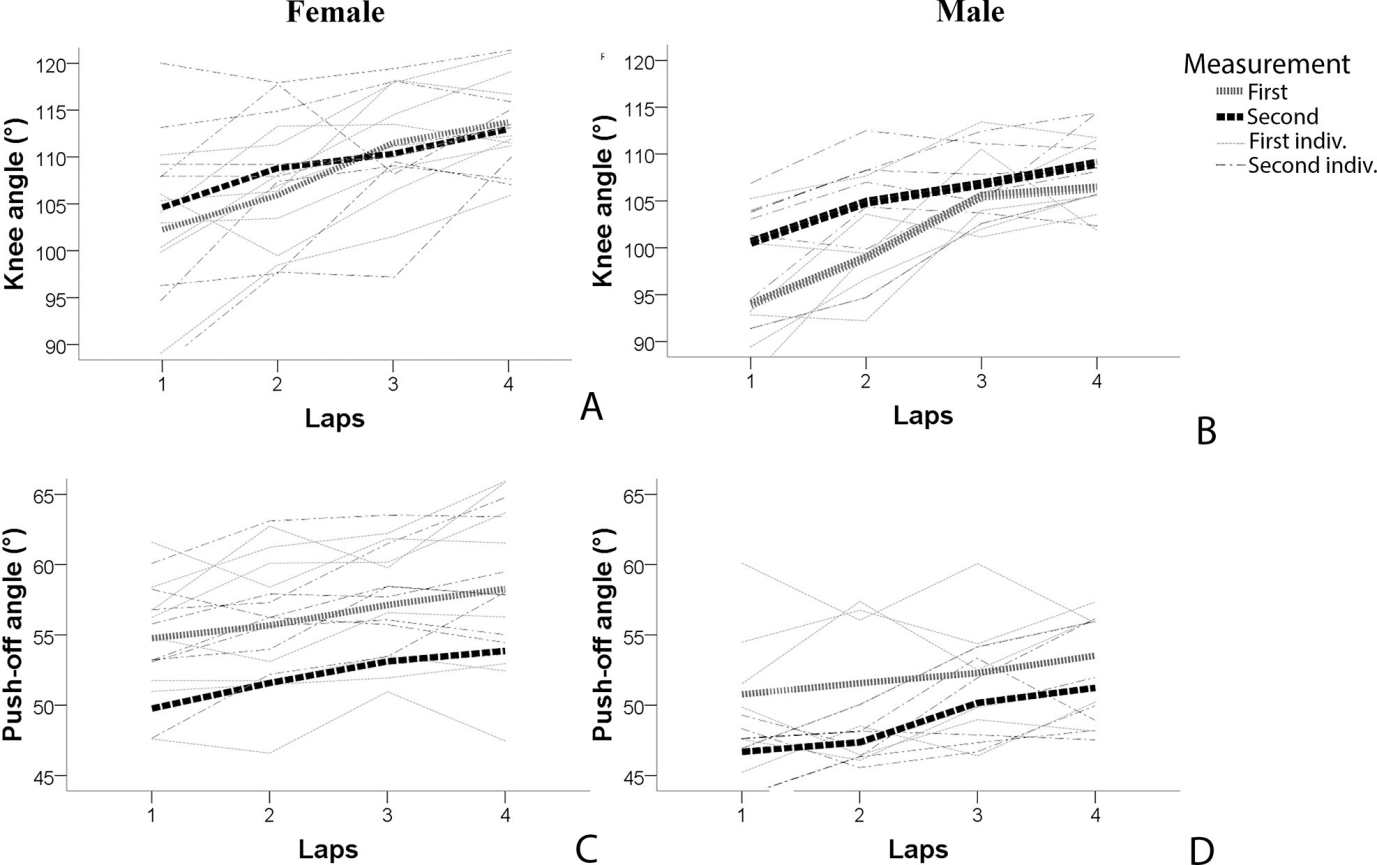
Changes in knee (A & B) and push-off (D & C) angles throughout the 1500 m for female (left) and male (right) elite junior speed skaters for the first measurement (grey line, mean age 17.3 yrs) and the second measurement 1.6±0.6 years later (black line, mean age 18.9 yrs).

For knee angles, a main effect for laps was found (F(3,39) = 53.793, p<0.001), with knee angles increasing from lap 1 to lap 2 (F(1,13) = 22.021, p<0.001), from lap 2 to lap 3 (F(1,13) = 22.644, p<0,001) and from lap 3 to lap 4 (F(1,13) = 9.397, p = 0.009), see Fig 3. No main effect for measurement was found (p = 0.216). An interaction effect of laps* measurements was found (F(3,39) = 3.241, p = 0.032), with less decay from lap 2 to 3 in the second measurement (F(1,13) = 6.295, p = 0.026). No interaction for laps*sex (p = 0.884) and laps*measurements*sex (= 0.968) were found. A between subject effect for sex was found (F(1,13) = 7.263, p = 0.018), with higher knee angles for female skaters. Average and individual changes in knee angles for female and male skaters can be seen in Fig 3A and 3B.

For push-off angles a main effect was found for laps (F(1.9,24.1) = 15.341, p<0.001), with push-off angles increasing from lap 2 to lap 3 (F(1,13) = 11.331, p = 0.005), and from lap 3 to lap 4 (F(1,13) = 5.101, p = 0.042). Push-off angles remained similar from lap 1 to lap 2 (p = 0.115). A main effect for the two measurements (F(1,13) = 5.340, p = 0.038) was also found, with smaller push-off angles in the second measurement than in the first measurement. No interaction effect were found (p<0.05). A between subject effect for sex was found (F(1,13) = 11.582, p = 0.005), with higher push-off angles for female skaters. Average and individual changes in push-off angles for female and male skaters can be seen in Fig 3C and 3D.

## Discussion

The aim of the present study was to investigate the profile of changes in technique throughout a 1500-m time-trial of elite junior speed skaters and investigates differences between sexes and performance levels. It additionally explored possible longitudinal development of technique over competitive seasons to provide perspectives on the development of elite junior speed skaters towards senior level.

### Cross-sectional data

The general profile for changes in knee angles for junior speed skaters was found to be an increase in body angles over the race. This was expected and similar to the increase in knee angles found in a previous article on 8 junior elite speed skaters [5]. In the present study it was additionally showed that knee angles in junior speed skaters increase every lap. Males showed smaller knee angles than females, but no differences between sexes for changes in knee angles were found. The better performing junior speed skaters had smaller knee angles compared to their less performing counterparts, though changes in knee angles over the race remained similar. The increase in knee angles can be considered as a deterioration of technique although various aspects play a role. The changes in knee angles might be a pacing like behavior to lessen the restriction of blood flow towards the working muscles throughout the race. A less crouched position, with higher knee angles, reduces the restriction of blood flow to the working muscles, but might simultaneously increase aerodynamic resistance [9, 10]. The trade-off between aerodynamic resistance and blood flow restriction was previously studied and showed to be neutralizing the effect of higher knee angles on velocity per lap [9]. It remains unknown whether at some point, lowering knee angles even further has a larger effect on the restriction of blood flow than on aerodynamic resistance. More detailed studies are needed to confirm this.

The general profile for changes in push-off angles for junior speed skaters was an increase in body angles over the race until 1100m, after which push-off angles remained similar. Males showed smaller push-off angles than females. Differences between sexes were found for changes of push-off angles, with male junior speed skaters showing a larger decay in the first three laps than female junior skaters. It might be that male juniors were more explosive in the beginning of the race, though this was not seen in their pacing behaviors (Table 1). As performance groups did not show different profiles of changes in technique, it is also not likely that the difference can be explained by differences in performance level. Therefore more research on differences in technique between female and male skaters is needed. The present study did show the importance of low push-off angles for performance, with smaller push-off angles for the faster performance group.

An additional finding of the present study was differences in pacing behavior between performance levels. A lower relative time spend on the 700-1100m section was found for the faster male skaters as well as for the faster female junior speed skaters (Table 1). This is in accordance with previous literature on male junior speed skaters [13], though the present study is the first to show this in female junior speed skaters as well. This is a relevant finding for future research and for speed skating practice. Differences in pacing behavior were hypothesized to be related with changes in technique, but it might be that our measurement frequency, once per lap, was not sufficient to find this relationship. Furthermore, it should be kept in mind that the present study used a basic method for capturing body angles which limits the accuracy level with respect to more advanced systems using body markers [22]. To ensure ecological validity and practical relevance the present study was done at official elite competition. As such, all included speed skaters were performing at one of the most important competition of the season, due to which a maximal effort can be expected to be given by all participants. A drawback of this choice is that using body markers was not possible. This impacted the ICC of our measurements, which was good for push-off angles, but moderate for knee angles. Caution was therefore taken in drawing conclusions on the knee angle data in the present study.

### Longitudinal data

The longitudinal analyses showed that over competitive seasons only push-off angles decreased. Knee angles remained similar between competitive seasons, but it appeared that junior speed skaters do develop the changes in knee angles within a race towards less decay from lap 2–3 in the second competitive season. No changes in the strategy of increasing push-off angles throughout the race appeared to be present. Whether the observed changes in knee and push-off angles can be explained by training or anthropometrics is still unknown as the present study did not obtain further background information about the individuals. Also the longitudinal results, should be interpreted with care, as only 15 skaters could be included. More longitudinal research including anthropometrical and training background information is needed to better understand the changes in technique over competitive seasons. Nevertheless the longitudinal observations can add to the interpretation of differences between junior and senior speed skaters.

### From junior to senior

For knee angles, differences between sexes were found over the 1500m race (female = 114.2°, male 109.4°). Previous literature on senior elite speed skaters showed no differences between sexes for knee angles (average 108° in the final three laps) at senior elite level [7]. Older studies did show higher average knee angles for female (122°) than male (111.6°) elite senior speed skaters [4, 16]. However, in the 80s, knee angles of female skaters were relatively high and it might be that female competition was not as evolved as male competition yet. With high-level performances at the world-level for females nowadays, it might well be that differences in knee angles between sexes are not present at the senior level anymore. This hypothesis is supported by the longitudinal results of the present study. The elite juniors appear not to develop towards smaller knee angles over 1.6 years. Maybe because they already adopt knee angles similar to senior elite skaters, which showed knee angles of around 108 degrees in the final three laps [7]. Adopting smaller knee angles might therefore be not beneficial for performance, when already low knee angles are adopted (Fig 3A and 3B). The only longitudinal difference found for knee angles was less decay from lap 2–3. Though only a small group (n = 15) was analyzed and the measurement of knee angles had a moderate ICC, this put some new perspectives on the technical development of speed skaters from junior to senior level. Noordhof et al. [7] found that senior speed skaters increase knee angles to 1100m, but maintained knee angles afterwards. The junior elites from the cross-sectional study showed an increase of knee angles in each lap. In sum, it might be that towards senior level the elite junior speed skaters do not adopt smaller knee angles to improve performance, but learn to maintain knee angles throughout the race and probably cope with the restriction of blood flow associated with low knee angles.

For push-off angles, differences between sexes were found over the 1500m race (female 53.9°, male 51.4°). Previous literature on senior elite speed skaters also showed differences between sexes for push-off angles [7]. Noordhof et al. [7] showed push-off angles for senior elite speed skaters around 55° in the final three laps. The junior athletes of the present study showed somewhat smaller angles. This is interesting as the longitudinal results suggest development to even smaller push-off angles. Whether this can be explained by not including the first lap, by combining both female and male athletes or by differences between junior and senior athletes is unknown. The cross-sectional results of the present study showed that junior speed skaters increase push-off angles from lap 1–3, while Noordhof [7] found that senior speed skaters increased push-off angles in the final three laps. The longitudinal results did not show different decay in push-off throughout the race to confirm these changes, but did show a development towards smaller push-off angles. It is therefore hypothesized that in order to improve performance towards senior level smaller push-off angles are beneficial from the beginning of the race onward, even when this means that push-off angles maintain increasing over the race. More research is needed to confirm these hypotheses on longitudinal development of knee and push-off angles.

### Practical guidelines

While keeping in mind its limitations (one measurement per lap and moderate to good ICC), the present study provides preliminary practical implications for trainers, coaches and their skaters aiming to improve their 1500-m time-trial performance. The results of the present study can be used to monitor technique as an underlying performance characteristic of performance development in junior speed skating. As male junior skaters showed smaller knee and push-off angles than female junior skaters, it is advised to make a distinction between sexes when analyzing their technique. Junior speed skaters can compare themselves with the profiles of changes in technique as well as with the pacing behaviors presented in the present study. For the development of technique towards the senior level in speed skaters, it might be better to focus on limiting the increase of knee angles during the midsection of the race. For push-off angles it seems to be more beneficial to focus on decreasing the push-off angle over the entire race.

## Conclusion

In elite junior speed skating, knee angles increase throughout the entire 1500-m time-trial, regardless of performance level and sex. Push-off angles increase from lap 1–3, regardless of performance level, but male skaters have a larger decay in these first three laps than female skaters. Faster skaters have smaller knee and push-off angles than slower skaters throughout the race. Differences in pacing profiles between performance groups were found, however no differences in the profile of changes in knee and push-off angles were found between performance groups. The longitudinal results suggests changes in technique over competitive seasons, with smaller push-off angles and better maintenance of knee angles in the midsection of the race at an older age. The results found in the present study emphasize the importance of studying junior and senior athletes separately, both for practical use and for understanding underlying mechanisms of performance.
